# Molecular and Functional Characterization of Wheat *ARGOS* Genes Influencing Plant Growth and Stress Tolerance

**DOI:** 10.3389/fpls.2017.00170

**Published:** 2017-02-08

**Authors:** Yue Zhao, Xuejun Tian, Yuanyuan Li, Liyuan Zhang, Panfeng Guan, Xiaoxia Kou, Xiaobo Wang, Mingming Xin, Zhaorong Hu, Yingyin Yao, Zhongfu Ni, Qixin Sun, Huiru Peng

**Affiliations:** State Key Laboratory for Agrobiotechnology and Key Laboratory of Crop Heterosis and Utilization, Beijing Key Laboratory of Crop Genetic Improvement, Department of Plant Genetics and Breeding, China Agricultural UniversityBeijing, China

**Keywords:** *TaARGOS*, transgenic gene, abiotic stress, ABA, wheat, *Arabidopsis*

## Abstract

Auxin Regulated Gene involved in Organ Size (ARGOS) is significantly and positively associated with organ size and is involved in abiotic stress responses in plants. However, no studies on wheat *ARGOS* genes have been reported to date. In the present study, three *TaARGOS* homoeologous genes were isolated and located on chromosomes 4A, 4B, and 4D of bread wheat, all of which are highly conserved in wheat and its wild relatives. Comparisons of gene expression in different tissues demonstrated that the *TaARGOSs* were mainly expressed in the stem. Furthermore, the *TaARGOS* transcripts were significantly induced by drought, salinity, and various phytohormones. Transient expression of the TaARGOS-D protein in wheat protoplasts showed that TaARGOS-D localized to the endoplasmic reticulum. Moreover, overexpression of *TaARGOS-D* in *Arabidopsis* resulted in an enhanced germination rate, larger rosette diameter, increased rosette leaf area, and higher silique number than in wild-type (WT) plants. The roles of *TaARGOS-D* in the control of plant growth were further studied via RNA-seq, and it was found that 105 genes were differentially expressed; most of these genes were involved in ‘developmental processes.’ Interestingly, we also found that overexpression of *TaARGOS-D* in *Arabidopsis* improved drought and salinity tolerance and insensitivity to ABA relative to that in WT plants. Taken together, these results demonstrate that the *TaARGOSs* are involved in seed germination, seedling growth, and abiotic stress tolerance.

## Introduction

An explosion in the global demand and consumption of agricultural crops for food, feed, and fuel, originating from the rapid increases in the global population and emerging economies, has made increased crop productivity a primary goal of breeding programs (Rosegrant and Cline, 2003; Edgerton, 2009). Crop yield improvements are either the result of increased total biomass production (larger plants tend to produce greater yields), an increased harvest index, or both (Quarrie et al., 2006; Fabre et al., 2016). Therefore, genes that control organ size and lead to higher yield are important targets of crop breeding (Gonzalez et al., 2009).

The ORGAN SIZE RELATED (OSR) family is known to promote plant organ growth and regulate the expression of organ size genes (Hu et al., 2003, 2006; Feng et al., 2011; Qin et al., 2014). Furthermore, previous data have shown that the OSR family is involved in the response to abiotic stresses (Guo et al., 2014; Rai et al., 2015; Shi et al., 2015, 2016a,b). In *Arabidopsis, AUXIN REGULATED GENE INVOLVED IN ORGAN SIZE* (*ARGOS*), the founding member of the *OSR* homologs, was identified as an auxin-induced gene that is expressed in developing organs. *AtARGOS* encodes a predicted integral membrane protein, and its overexpression is sufficient to increase organ size by stimulating cell proliferation; conversely, down-regulation causes reduced organ growth. *AtARGOS* promotes plant organ growth mainly by enhancing the continuous expression of *ANT* and *CycD3;1* (Hu et al., 2003). An earlier study indicated that the *AtARGOS* homolog *ARGOS-LIKE* (*AtARL*) also regulates lateral organ size. Increased *AtARL* expression is sufficient to cause organ enlargement due to an increase in cell size, while reduced *AtARL* expression leads to smaller organs with less expanded cells. In contrast to *AtARGOS* mediation of auxin effects on growth, *AtARL* was hypothesized to function downstream of brassinosteroids (BR; Hu et al., 2006). There are four OSR homologs in *Arabidopsis*, and other members of this family (*OSR1* and *OSR2*) are also known to promote plant organ growth when overexpressed in *Arabidopsis* (Feng et al., 2011; Qin et al., 2014). In rice, five OSR members were identified including *OsARGOS* (Feng et al., 2011). It was found that overexpression of *OsARGOS* in *Arabidopsis* increases organ growth as well (Wang et al., 2009). The *ARGOS* gene exists as eight copies in maize. Overexpression of *ZmARGOS1* in maize enhances maize organ growth and increases yield (Guo et al., 2014). Furthermore, maize *ARGOSs* have been found to be related to abiotic stress (Guo et al., 2014; Rai et al., 2015; Shi et al., 2015, 2016a,b). For example, overexpression of *ZmARGOS1* and *ZmARGOS8* significantly improves tolerance to drought stress through an ethylene-dependent regulation pathway in *Arabidopsis* and maize. Additionally, maize plants overexpressing *ZmARGOS8* exhibit a greater grain yield than WT controls under both drought stress and well-watered conditions (Shi et al., 2015). Recently, Shi et al. (2016a) demonstrated that ZmARGOS1 and ZmARGOS8 modulate ethylene signal transduction through REVERSION-TO-ETHYLENE SENSITIVITY1-LIKE (RTL) proteins (Shi et al., 2016a). However, in wheat (*Triticum aestivum* L. 2*n* = 6*x* = 42, genomes AABBDD), an important food crop around the world, no *ARGOS* genes have been reported.

In the current study, we report the identification, physical localization, and expression patterns of *ARGOS* homoeologous genes in bread wheat. Additionally, the subcellular localization of TaARGOS-D was determined, and the effects of overexpression of *TaARGOS-D* in *Arabidopsis* on plant growth and stress tolerance were studied.

## Materials and Methods

### Plant Materials and Stress Treatments

Wheat cv. Jingdong6 was used for gene cloning and expression analyses. Seeds were sterilized in a 1% NaClO solution and incubated for 1 day in sterile distilled water at 22°C in the dark. After germination, the seedlings were grown in a greenhouse (22°C, 16 h photoperiod). For drought, salt, and exogenous hormone treatments, 7-day-old seedlings were transferred to a water solution containing 20% PEG6000, 200 mM NaCl, 200 μM ABA, 100 μM methyl jasmonate (MeJA), 50 μM α-naphthaleneacetic acid (NAA), 50 μM aminocyclopropane-1-carboxylic acid (ACC, an ethylene precursor), 50 μM gibberellic acid (GA_3_), or 10 nM BR. All treatments were performed under the same conditions for 0, 1, 2, 4, 6, 12, and 24 h. After exposure to stress, the leaves were immediately frozen in liquid nitrogen prior to expression analysis. To study the tissue-specific expression of the *TaARGOSs*, dry seeds, seedlings at 1–6 days after germination (DAG), and roots, stems, leaves, and young spikes (YS) in the booting stage were obtained from Jingdong6 wheat. Nine diploid progenitor species [three A genome *T. urartu* species, three S (possibly modified B) genome *Aegilops speltoides* species, and three D genome *A. tauschii* species], three tetraploid *T. dicoccum* species, and six common wheat cultivars were used for the sequence comparison analysis (Supplementary Table S1). Chinese Spring (CS) nulli-tetrasomic (NT) lines were employed to determine the chromosomal locations of each gene.

The *Arabidopsis* Columbia-0 ecotype was used as the WT. Seeds were surface sterilized with a 5% NaClO solution and cold treated at 4°C for 3 days in the dark, then plated on Murashige and Skoog (MS) medium containing 3% (w/v) sucrose and 0.8% agar. Seven-day-old seedlings were transferred to a growth chamber under 16/8 h light/dark conditions at 22°C.

### Cloning of the TaARGOSs and Sequence Analysis

The sequence of the *Arabidopsis ARGOS* gene was used as probe for BLAST searches against the wheat genome sequence database of the International Wheat Genome Sequencing Consortium (IWGSC)^1^ and the UniProt databases^2^. Based on the nucleotide sequence polymorphisms of the *TaARGOSs*, the genome-specific primer sets TaARGOS-AF1/TaARGOS-AR1, TaARGOS-BF1/TaARGOS-BR1, and TaARGOS-DF1/TaARGOS-DR1 were used to obtain the full-length DNA sequences (including the 5′ and 3′ flanking regions) from chromosomes 4A, 4B, and 4D, respectively. Each primer set was tested with CS NT lines to determine the chromosomal locations of the amplified genes. All PCR primers used in this study are described in Supplementary Table S2.

Genomic DNA was isolated from young leaves using the CTAB method. The PCR assays were performed using high-fidelity Primestar polymerase (TaKaRa, Dalian, China) under the following conditions: 98°C for 3 min, followed by 35 cycles of 98°C for 15 s, 58°C for 15 s, and 72°C for 90 s, with a final extension at 72°C for 3 min. The PCR products were recovered, cloned into the pMD18-T vector (TaKaRa, Dalian, China) and sequenced. Total RNA was extracted using the TRIzol reagent according to the manufacturer’s protocol (TianGen, Beijing, China) and treated with DNase I to remove the contaminating genomic DNA. Two micrograms of total RNA were reverse transcribed using AMV Reverse Transcriptase (TaKaRa, Dalian, China) following the manufacturer’s instructions. The cDNA was diluted and used as a template for RT-PCR or quantitative RT-PCR (qRT-PCR). According to the acquired genomic sequences, gene-specific primer sets were designed to amplify the *TaARGOS* cDNA sequences from wheat cv. Jingdong6. The PCR conditions and sequencing procedures were as described above.

The primers employed for PCR were designed using DNAMAN software. Alignment analysis was performed with ClustalW. Phylogenetic trees were constructed with MEGA6 software using the neighbour-joining (NJ) method (Tamura et al., 2013).

### Gene Expression Analysis

Real-time qRT-PCR analysis was conducted using SYBR Premix EX Tag^TM^ (TaKaRa, Dalian, China) in a volume of 20 μl in a Bio-Rad CFX96 real-time PCR detection system^3^. The PCR parameters were as follows: 94°C for 3 min, followed by 40 cycles of 94°C for 15 s, 60°C for 20 s, and 72°C for 20 s. The wheat *β-actin* gene was used as a reference gene (Hu et al., 2013). Each amplification reaction was repeated three times. Validation experiments were performed to demonstrate that the amplification efficiency of the *TaARGOS*-specific primers was approximately equal to the amplification efficiency of the endogenous reference primers. Quantification of the target gene expression was carried by the comparative CT method (Livak and Schmittgen, 2001). The primers employed for qRT-PCR are listed in Supplementary Table S2.

For the semi-qRT-PCR assay, the PCR conditions for the amplification of *TaARGOS-D* were as follows: 5 min at 94°C, followed by 34 cycles of 20 s at 94°C, 20 s at 60°C, and 20 s at 72°C. The same conditions were used for the amplification of *Actin2* of *Arabidopsis*, except that the number of PCR cycles was decreased to 25.

### Subcellular Localization Analysis

The full-length coding sequence of *TaARGOS-D* without the stop codon was obtained through PCR amplification with high-fidelity Primestar polymerase and primers with restriction enzyme sites (*Hind*III and *Xba*I). The double-digested PCR products were inserted into the N-terminus of the *green fluorescent protein* (*GFP*) gene under the control of the cauliflower mosaic virus (CaMV) 35S promoter. The construct was confirmed by sequencing and transformed into wheat protoplasts via the PEG-mediated transformation method (Yoo et al., 2007; Shan et al., 2014). The transformed wheat protoplasts were cultured at 25°C for 18 h and observed under a confocal microscope (Eclipse TE2000, Nikon).

### GUS Histochemical Staining

The *TaARGOS-D* promoter-*GUS* reporter plasmid was constructed by cloning a PCR-amplified DNA fragment containing the *TaARGOS-D* 5′ sequence (1670 bp) into the GUS reporter plasmid pCAMBIA1381Z. Histochemical GUS staining was performed as described previously (Jefferson et al., 1987). Whole transgenic *Arabidopsis* seedlings or different tissues were immersed in staining buffer at 37°C in the dark from 3 h to overnight depending on the experimental requirements. For stress treatment, the 7-day-old transgenic seedlings were transferred to one of the following MS plates: 10% PEG6000, 150 mM NaCl, 100 μM ABA, 10 μM MeJA, 50 μM NAA, 10 μM ACC, or 50 μM GA for 6 h before histochemical staining.

### Construction of Transgenic Plants

The full-length coding region of *TaARGOS-D* cDNA was amplified through PCR using gene-specific primers containing attB sites (Supplementary Table S2, the attB sites are underlined). The PCR product was cloned into the Gateway plant expression vector pB2GW7 harboring the CaMV 35S promoter. The construct was then sequenced and introduced into *Agrobacterium* GV3101 and transformed into *Arabidopsis* plants via the floral dip method (Clough and Bent, 1998). The positive transgenic plants were screened and then further identified through RT-PCR.

### Microscopy Counts of Leaf Epidermal Cells

The fifth leaf was collected from greenhouse-grown 3-week-old plants. Chlorophyll was removed by washing the leaf 2∼3 times until the washing solution (70% ethanol) remained clear. The leaves were then soaked in Hoyer’s solution to make the tissue transparent. Next, a 1-cm^2^ section was removed from the midpoint of these fixed leaves, cleared in a saturated solution of chloral hydrate, and mounted on a glass slide under a cover glass. Images were collected using a Nikon Ti-U microscope. All whole cells captured in the field were counted.

### RNA-Seq Analysis

RNA-seq analysis was carried out using an *Arabidopsis* transgenic line (L3). Total RNA was isolated from the 6-day-old seedlings of L3 and WT plants grown in Petri dishes under normal conditions using the TRIzol reagent (TianGen, Beijing, China). cDNA synthesis was performed according to the previously described method. Paired-end (PE) sequencing libraries were prepared using the TruSeq RNA Sample Preparation Kit v2 (Illumina, San Diego, CA, USA). Sequencing was performed on the Illumina HiSeq 2000 platform. Raw reads from the libraries were filtered, and the clean reads from each library were then mapped to the reference *Arabidopsis* genome (TAIR 10) using bowtie2 (Langmead and Salzberg, 2012). The reliability of the RNA-seq data was confirmed by calculating Pearson’s correlation coefficient between each pair of biological replicates. Gene expression levels were measured based on RPKM (reads per kilobase of exon model per million mapped reads) values. Differentially expressed genes (DEGs) in the six libraries from wild-type and L3 plants (three libraries from three biological replicates for each sample) were identified according to false discovery rate (FDR) values ≤0.05 and fold change values ≥2. Pathway and Gene Ontology (GO) analyses were performed using the agriGO database^4^.

### Stress Treatment Analysis of Transgenic *Arabidopsis* Plants

For the germination assays under stress treatment, approximately 64 seeds from each of the transgenic lines and WT plants were surface-sterilized and then planted on MS agar medium containing either 1 μM ABA, 10% PEG6000, or 100 mM NaCl. The data were collected every day, and photographs were taken on the 7th day. Germination was defined as a clear sign of the emergence of the radicle tip. The germination analyses were repeated three times.

For the salt and drought stress treatments, surface-sterilized transgenic and WT seeds were planted on MS medium plates and grown under standard culture conditions with white fluorescent light (16 h light/8 h dark) at 22°C. Seven-day-old seedlings were transferred to MS medium containing either 0 or 10% PEG6000 or 150 mM NaCl and then planted vertically for 5 days, after which the root lengths and fresh weights of the transgenic and WT plants were measured and photographed. Additionally, 7-day-old seedlings were transferred to mixed soil (rich soil: vermiculite = 2: 1, v/v). Each pot contained four seedlings, which were grown for 2 weeks with sufficient watering. The plants were then subjected to drought stress treatment by withholding irrigation for 2 weeks. For salt stress analysis, the transgenic and WT plants were irrigated with a 200 mM NaCl solution once every 3 days for 2 weeks. The results were calculated based on three independent experiments.

## Results

### Isolation and Chromosomal Location of *TaARGOS* Homoeologous Genes

**FIGURE 1:**
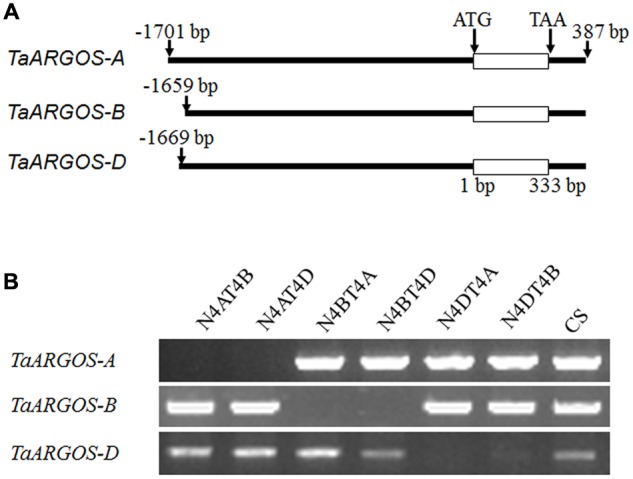
**Cloning regions of the *TaARGOSs* using genome-specific primers (A)** and each primer set was tested on CS nulli-tetrasomic lines **(B)**. Three sets of primers recognized as *TaARGOS-4A, -4B*, and *-4D* on chromosomes 4A, 4B, and 4D, respectively. N4AT4B: nullisomic 4A-tetrasomic 4B; N4AT4D: nullisomic 4A-tetrasomic 4D; N4BT4A: nullisomic 4B-tetrasomic 4A; N4BT4D: nullisomic 4B-tetrasomic 4D; N4DT4A: nullisomic 4D-tetrasomic 4A; N4DT4B: nullisomic 4D-tetrasomic 4B; *CS*: Chinese Spring.

**FIGURE 2:**
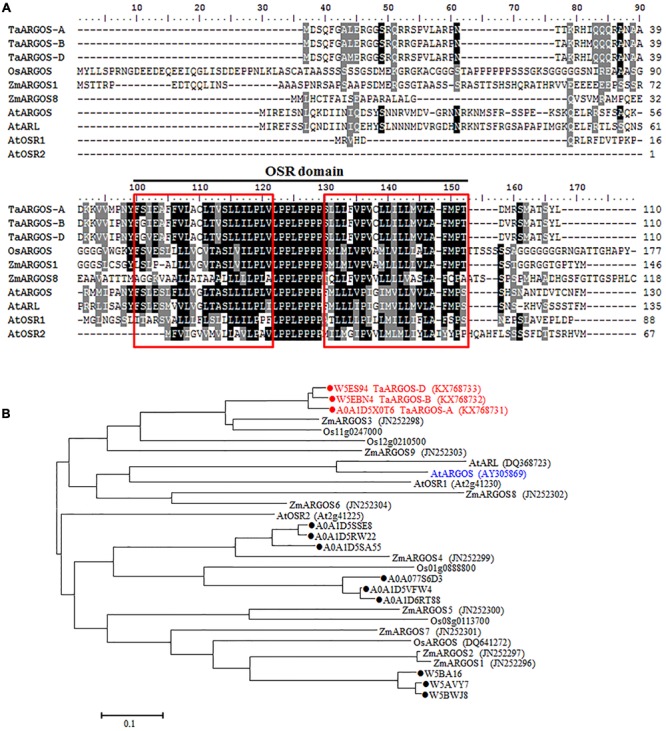
**Sequence alignment and phylogenetic analysis of the TaARGOSs and other ARGOS proteins. (A)** Amino acid sequence alignment of the TaARGOSs and the ARGOS from *Arabidopsis* AtARGOS (Hu et al., 2003), AtARGOS-like (Hu et al., 2006), AtOSR1 (Feng et al., 2011), and AtOSR2 (Qin et al., 2014), rice OsARGOS (Wang et al., 2009), and maize ZmARGOS1 (Guo et al., 2014) and ZmARGOS8 (Shi et al., 2015). Consensus sequences (100 and 75%) are exhibited in black and gray shading. The black line represents the conserved OSR domain. Red boxes indicate the putative transmembrane helices. **(B)** Phylogenetic tree based on amino acid sequences indicating the relationships of TaARGOSs with other plant ARGOS proteins. The accession numbers of the amino acid sequences are given in brackets. Twelve wheat protein sequences from the UniProt databases were indicated by dots. ZmARGOS2 is an allele variant of the ZmARGOS1 gene.

Based on the sequence of the *Arabidopsis ARGOS* gene, three wheat contig sequences (*T. aestivum* 4AL contig 346934, *T. aestivum* 4BS contig 186453, and *T. aestivum* 4DS contig 18687) were identified in the IWGSC databases. According to the observed BLAST hits, we designed genome-specific primer sets to obtain *TaARGOS* homoeologous genes from the A, B, and D genome sequences (**Figure 1A**). Each primer set was tested in CS NT lines. Amplification products were observed in four CS NT lines, with the exception of NT4A4B and NT4A4D, indicating that the *TaARGOS* amplified by the TaARGOS-AF1/TaARGOS-AR1 primer set was located on chromosome 4A, and this gene was designated *TaARGOS-A* (KX768731). Likewise, *TaARGOS-B* (KX768732) and *TaARGOS-D* (KX768733), amplified by primer sets TaARGOS-BF1/TaARGOS-BR1 and TaARGOS-DF1/TaARGOS-DR1, were located on chromosomes 4B and 4D, respectively (**Figure 1B**). To obtain the cDNA sequences of these genes, we designed gene-specific primers to amplify the genes from the cDNA of wheat leaves. By comparing the genomic and cDNA sequences, we found that *TaARGOS-A, TaARGOS-B*, and *TaARGOS-D* consisted of one exon and contained a 333-bp open reading frame encoding 110 amino acid residues (**Figure 2A**). Multiple sequence alignments using DNAMAN revealed that the deduced polypeptides of the three TaARGOS homoeologous proteins shared 98.0% sequence similarity. In addition, we found that all three TaARGOS proteins contained an organ size-related (OSR) conserved domain in the C-terminal half of the protein, consistent with ARGOS proteins from various other species (**Figure 2A**).

**FIGURE 3:**
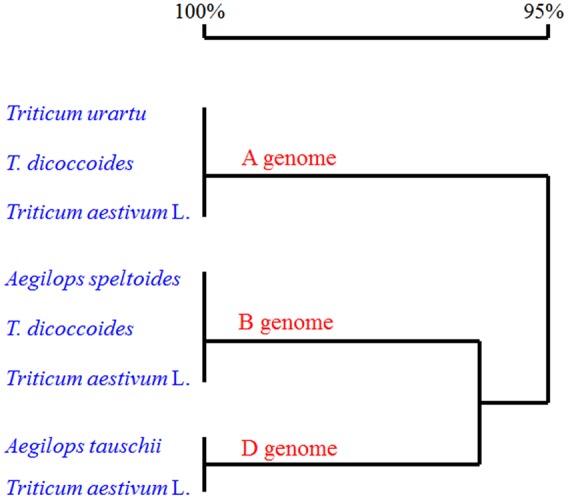
**Alignment of deduced amino acid sequences of three *TaARGOS* homoeologous genes in wheat and its relatives.** The coding sequences of the *TaARGOSs* in three A genome *T. urartu* species, three B genome *Aegilops speltoides* species, three D genome *A. tauschii* species, three tetraploid *T. dicoccoides* species, and six common wheat cultivars (*T. aestivum* L.) were used for sequence analysis. Homology tree was constructed by DNAMAN 7 software.

We also searched the wheat OSR genes against the UniProt databases using the OSR domain sequence of the *Arabidopsis* ARGOS, and 12 uncharacterized wheat protein sequences were identified. Three out of the 12 identified sequences were the ones we cloned. Phylogenetic analysis of the deduced amino acid sequences with known OSR proteins in rice and *Arabidopsis* showed that the TaARGOSs were homologous to *Arabidopsis* AtARGOS, which were grouped into the same clade (**Figure 2B**). We next isolated and sequenced the genomic sequences of the *TaARGOSs* and their orthologs in diploid, tetraploid and hexaploid wheat. The *TaARGOSs* (including the coding and partial flanking regions) were amplified from 12 progenitor accessions and 6 modern hexaploid cultivars (Supplementary Table S1) using genome-specific primers. All sequences identified in hexaploid wheat were present in the corresponding genome donor species, and the coding regions exhibited no nucleotide differences in the A, B, and D genomes (**Figure 3**), which suggested that *TaARGOSs* are highly conserved during the polyploidization.

### Expression Analysis of *TaARGOS* Genes in Wheat

**FIGURE 4:**
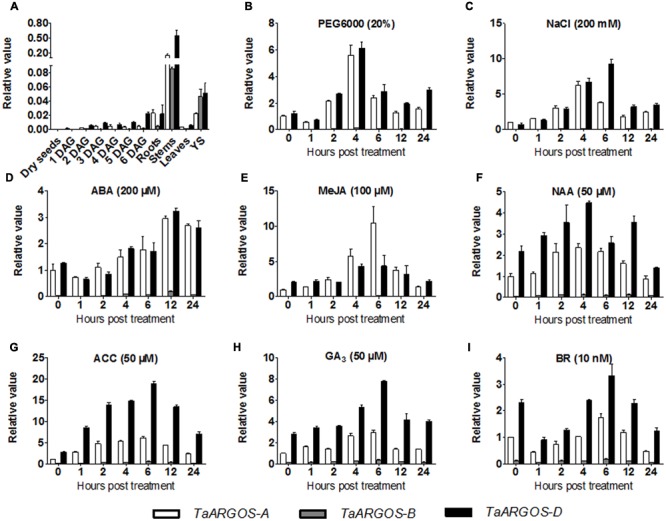
**Expression pattern of the *TaARGOS* genes. (A)** Expression pattern analysis by qRT-PCR of the three *TaARGOS* homoeologous genes in various wheat tissues. Total RNA was isolated from roots, stems, leaves, young spikes (YS), dry seeds, and seedlings at 1–6 days after germination (DAG). **(B–I)** qRT-PCR assessment of expression patterns of the wheat *TaARGOS* genes under PEG, NaCl, and exogenous ABA, MeJA, NAA, ACC, or GA_3_ treatments. Total RNA was isolated from leaves of wheat seedlings. The *β-actin* gene was used as an internal reference. Error bars show standard deviation (SD) of three independent biological replicates.

To understand the temporal-spatial expression pattern of the *TaARGOS* homoeologous genes, we analyzed the expression patterns of the *TaARGOSs* in different wheat tissues. qRT-PCR analysis showed that the transcription levels of the *TaARGOSs* were different between tissues. The maximal expression levels of the *TaARGOSs* were detected in the stem at the booting stage, while moderate expression levels were detected in YS; very low expression levels were detected at early seed germination; and no expression was detected in dry seeds (**Figure 4A**). Interestingly, different expression patterns were observed for *TaARGOS-A, TaARGOS-B*, and *TaARGOS-D*, suggesting that these homeologs may play multiple roles at different developmental stages. *TaARGOS-D* showed the highest expression level in all of the tested tissues (**Figure 4A**), indicating that it had a greater impact on regulating plant development than the other two homeologs. Thus, the *TaARGOS-D* gene was selected for further functional analysis.

Previous studies demonstrated that the *Arabidopsis* and maize *ARGOS* genes enhance plant drought tolerance and can be induced by various hormones (Qin et al., 2014; Shi et al., 2015). To determine whether the *TaARGOSs* function in stress responses in wheat, we assayed their expression following exposure of the plants to PEG, NaCl, ABA, MeJA, NAA, ACC, GA_3_, and BR. As shown in **Figure 4**, the *TaARGOSs* were induced but were differentially expressed in response to abiotic stresses and hormones. Under PEG stress, the expression of the *TaARGOSs* peaked after 4 h (**Figure 4B**). Under salt stress, the expression of *TaARGOS-A* and *TaARGOS-D* increased and peaked at 4 and 6 h, respectively, after which the level of mRNA accumulation gradually declined (**Figure 4C**). Following exogenous ABA treatment, the expression of the *TaARGOSs* was up-regulated at 4 h and peaked at 12 h (**Figure 4D**). Upon exogenous MeJA treatment, the expression of *TaARGOS-A* peaked rapidly at 6 h and then declined rapidly and reached pretreatment levels at 24 h; however, *TaARGOS-D* expression was up-regulated at 4 h (**Figure 4E**). Under NAA treatment, the expression of the *TaARGOSs* increased gradually and peaked at 4 h (**Figure 4F**). The expression of the *TaARGOSs* was gradually up-regulated after exposure to exogenous ACC or GA_3_ and peaked at 6 h (**Figures 4G,H**). The expression of the *TaARGOSs* decreased after treatment with exogenous BR and reached pretreatment levels after 4 h (**Figure 4I**).

### Promoter Isolation and Activity Analysis of *TaARGOS-D*

**FIGURE 5:**
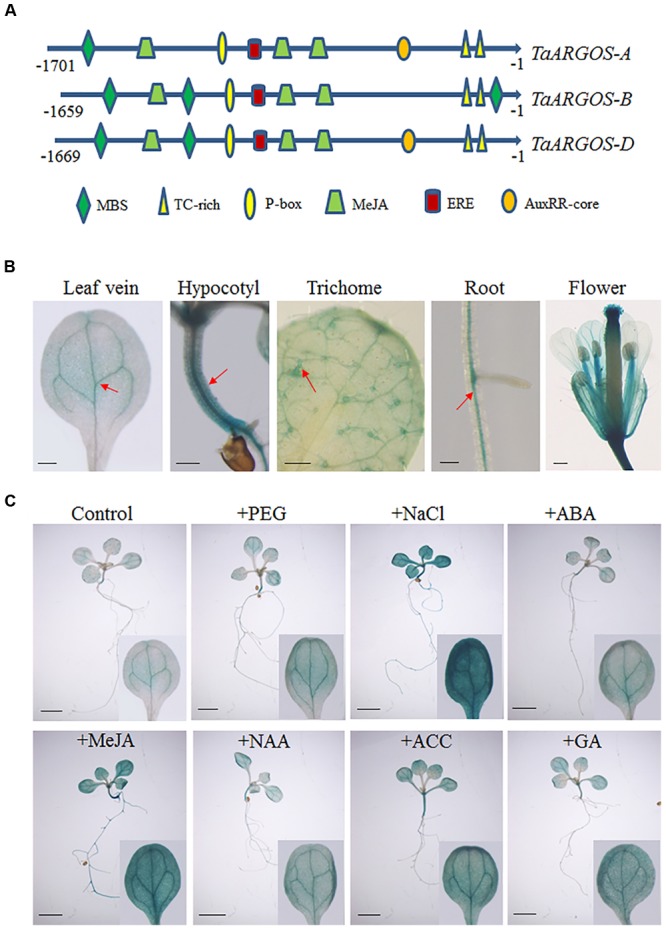
**Promoter isolation and activity analysis of the *TaARGOSs* (A)** *Cis*-acting regulatory elements of promoter regions of *TaARGOSs*. **(B)** GUS staining of various *pTaARGOS-D*::*GUS* transgenic *Arabidopsis* tissues. GUS staining is mainly localized to leaf veins, hypocotyl, the trichomes of the first true leaves, roots, and flower (arrow). Scale bars = 0.2 mm. **(C)** 7-day-old *Arabidopsis pTaARGOS-D*::*GUS* seedlings were grown on MS plates before transfer to either MS plates (Control), plates with 10% PEG6000, 150mM NaCl, 100 μM ABA, 10 μM MeJA, 50 μM NAA, 10 μM ACC, or 50 μM GA3 and treated for 6 h. Enhanced GUS staining is visible in stresses treated seedlings. Scale bars = 2 mm.

To gain further insight into the mechanism responsible for the transcriptional regulation of the *TaARGOSs*, we isolated the sequence 1659–1701 bp upstream of the start codons of the three *TaARGOSs* using a PCR strategy. We searched for putative *cis*-acting elements in the promoter regions using PlantCARE^5^ (**Figure 5A**). A number of regulatory elements responding to defence and stress were recognized, including MBS and TC-rich binding sequences. In addition, an auxin-responsive element (AuxRR-core), a gibberellin-responsive element (P-box), an ethylene-responsive element (ERE), and an MeJA-responsive element (CGTCA-motif) were identified. The promoter region of *TaARGOS*-*B* was distinct from that of *TaARGOS*-*A* and *TaARGOS*-*D*. Compared with *TaARGOS*-*A* and *TaARGOS*-*D*, the auxin-responsive element was deleted at the same position in the promoter region of *TaARGOS*-*B* (**Figure 5A**).

To further study the expression pattern of the *TaARGOSs*, we prepared the *pTaARGOS-D::GUS* construct, with the *GUS* reporter gene driven by the *TaARGOS-D* promoter, and transferred it into *Arabidopsis*. GUS staining of transgenic *Arabidopsis* revealed that the *TaARGOS-D* promoter is very active in the leaf veins, hypocotyl, the trichomes of the first true leaves, roots and flowers (**Figure 5B**). Stress-responsive expression of *TaARGOS-D* was further confirmed by histochemical staining in 7-day-old seedlings expressing the *pTaARGOS-D::GUS* construct and treated with either 10% PEG6000, 150 mM NaCl, 100 μM ABA, 10 μM MeJA, 50 μM NAA, 10 μM ACC, or 50 μM GA_3_ for 6 h in MS agar medium. As shown in **Figure 5C**, enhanced GUS activity was observed in NaCl-, PEG- and exogenous hormone-treated seedlings compared with untreated controls.

### TaARGOS-D Localizes to the Endoplasmic Reticulum

**FIGURE 6:**
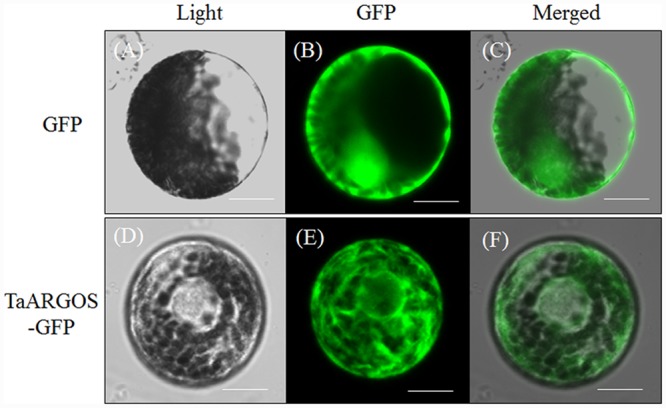
**Subcellular localization of the TaARGOS protein in wheat protoplasts.** Photographs were taken in the light field (**A** and **D**), in the dark field for green fluorescence (**B** and **E**), and in combination for morphology of the cells (**C** and **F**). Bars = 10 μM.

To determine the subcellular localization of the TaARGOS proteins in wheat cells, we transiently expressed the 35S::TaARGOS-D-GFP fusion protein in wheat protoplasts. As shown in **Figure 6**, the green fluorescence signal of the control 35S::GFP was uniformly distributed throughout the cell (upper panel), whereas TaARGOS-D-GFP exhibited a network structure (lower panel) consistent with the ER-localized GFP signals from the GFP: ARGOS and eGFP:MRN1 constructs (Go et al., 2012; Rai et al., 2015), indicating that TaARGOS-D localizes to the ER.

### Transgenic Overexpression of *TaARGOS-D* Increases Plant Growth

**FIGURE 7:**
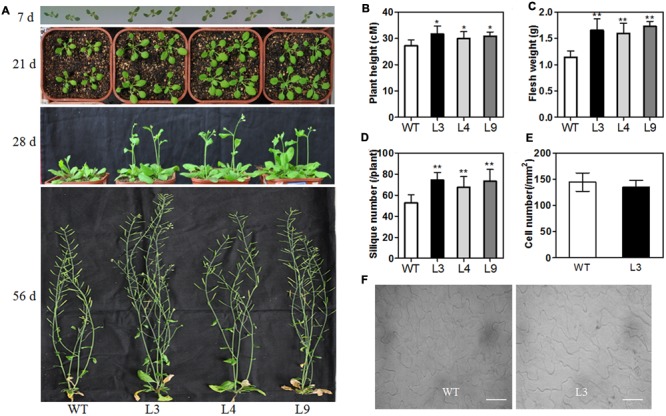
**Morphological differences between WT and transgenic line at different developmental stages. (A)** Phenotype of WT and overexpressing *TaARGOS-D* seedlings grown for 7, 21, 28, and 56 days, respectively. Plant height **(B)**, fresh weight **(C)**, and silique number **(D)** of 56-day-old WT and *TaARGOS-D* transgenic plants (*n* = 10). **(E,F)** Number of epidermal cells per area in the adaxial surface of fully expanded fifth rosette leaves of transgenic line and WT plants (*n* = 5). Bars = 50 μM. Error bars represent the means ± SD. Statistical significance was determined by a Student’s *t*-test **(B–E)**; significant differences (*P* ≤ 0.05) are indicated by asterisk.

To further understand the role of the *TaARGOSs* in plant growth, we generated transgenic *Arabidopsis* plants overexpressing *TaARGOS-D* under the control of the CaMV 35S promoter. RT-PCR was performed to assess the expression of *TaARGOS-D* in 11 T3 homozygous transgenic lines, and three independent lines with high expression levels (L3, L4, and L9) were used for further analysis (Supplementary Figure S1). In the germination process, the seed germination of the three transgenic lines was significantly faster than that of the WT line (Supplementary Figure S2), with 90% of the seeds of the transgenic lines germinating in the 36 h after sowing, while only approximately 70% of the WT seeds germinated during the same time period. At the seedling stage, the transgenic plants initially grew larger than the WT plants, but the difference decreased gradually after the bolting stage, and the height of the transgenic plants was eventually only slightly greater than that of WT plants (**Figures 7A,B**). The fresh weight of the transgenic plants was markedly higher than that of the WT plants at the mature period (**Figure 7C**). A similar difference between transgenic lines and WT plants was observed in the number of siliques per plant (**Figure 7D**). Although silique size was indistinguishable, earlier bolting and flowering were observed in transgenic plants (**Figure 7A**). Thus, these data demonstrate that the observed enhancement of plant growth was due to a faster growth rate rather than an extended growth period.

Since the transgenic plant seedlings displayed large leaves, the adaxial side of the 5th leaves of 21-day-old WT and L3 plants was visualized using a Nikon Ti-U microscope, which was subsequently employed to measure the cell number in the leaves (**Figures 7E,F**). We counted the leaf epidermal cell number per unit area and the cell number within 1 mm^2^. The average number of cells in the transgenic lines was found to be the same as in the WT line, indicating that cell size was unchanged in the transgenic plants. The L3 leaves were approximately twice as big as the WT leaves. Therefore, we believe that the changes in overall plant size were due to a change in cell numbers rather than cell size. Similar mechanisms have been reported for *Arabidopsis ARGOS* gene (Hu et al., 2003).

### Transgenic Overexpression of *TaARGOS-D* Affects Development-Related Genes

To investigate the possible molecular mechanisms underlying the effect of the *TaARGOSs* on plant growth, we used the RNA-seq approach to identify genes showing altered expression levels in the *TaARGOS-D* transgenic plants. Processing RNA samples using the Illumina HiSeq 2000 system yielded more than eight million reads, each 125 bp in length, encompassing 2 Gb of sequence data for each sample, which were then mapped to the reference genome (Supplementary Figure S3A). The Pearson’s correlation coefficients obtained by comparing three biological replicates of the either the WT or transgenic lines (L3), visualized in the correlation plot (Supplementary Figure S3B), are close to 1. These analyses demonstrated that the raw RNA-seq data exhibited high levels of reliability and reproducibility.

**FIGURE 8:**
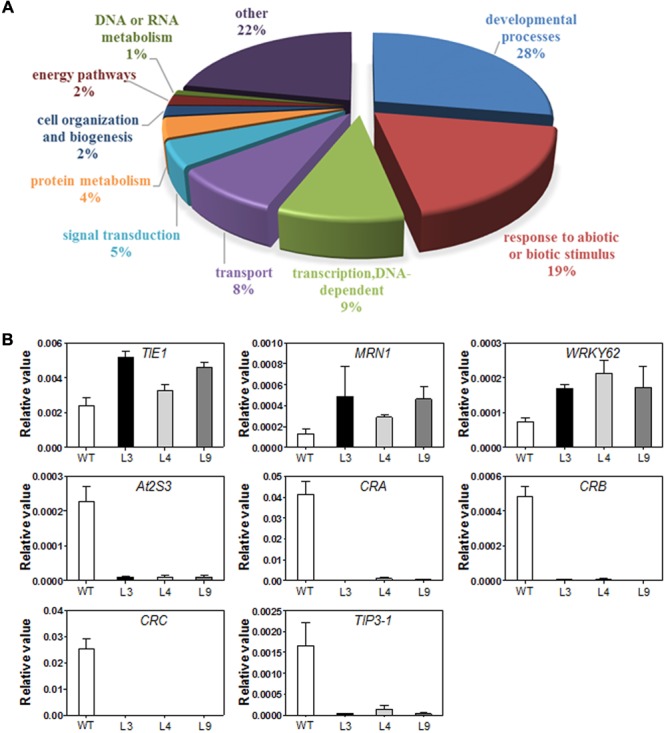
**(A)** Gene ontology classification for differentially expressed genes (DEGs) in WT and *TaARGOS-D* transgenic plants. **(B)** DEGs in *TaARGOS-D* transgenic and WT *Arabidopsis* plants by qRT-PCR analysis. Error bars show SD of three replications.

The statistical analysis identified a total of 105 DEGs between the transgenic and WT plants using a *Q*-value < 0.05 as a cutoff, among which 35 (33%) were up-regulated, and 70 (67%) were down-regulated, with fold changes higher than 2. Not surprisingly, growth and development genes dominated the list (**Figure 8A**, Supplementary Table S2). *AtTIE1* encodes an EAR motif-containing protein that controls leaf development by affecting cell differentiation (Tao et al., 2013). In addition, we detected two operon-like gene clusters that were also up-regulated in transgenic plants, which have been shown to be critical for growth and development in *Arabidopsis* (Field and Osbourn, 2008; Field et al., 2011; Go et al., 2012). The first operon-like gene cluster is essential for the synthesis of thalianol-derived triterpenes (the thalianol cluster), and consists of four contiguous genes, *THAS1, CYP708A2, CYP705A5*, and *ACT*. The second operon-like triterpene cluster (the marneral cluster) comprises the *MRN1, CYP71A16*, and *CYP705A12* genes.

Consistent with the increased growth rate of the transgenic seedlings, several seed storage protein (SSP) genes exhibited markedly reduced expression in the transgenic seedlings compared with the WT plants (Supplementary Table S3), including three 12S SSP-encoding genes (*CRA1, CRB*, and *CRC*) and four 2S SSP-encoding genes (*At2S1, At2S2, At2S3*, and *At2S5*). A few ABA-related genes were also markedly inhibited in the transgenic seedlings, such as the tonoplast intrinsic proteins *TIP3-1* and *EM1*. In addition, a number of bHLH transcription factor genes, such as *bHLH38, bHLH39, bHLH100*, and *bHLH101*, which have been associated with iron-deficiency responses and leaf morphogenesis development, were transcriptionally inhibited in the transgenic plants.

To validate the expression profiles obtained via RNA-seq, 6-day-old seedlings of Col-0 and three transgenic lines (L3, L4, and L9), grown under the same conditions used for RNA-seq, were collected for RNA extraction and qRT-PCR analysis. Eight genes selected from Supplementary Table S3 were examined, and the results were in agreement with the RNA-seq data (**Figure 8B**).

### Transgenic *Arabidopsis* Plants were Less Sensitive to Exogenous ABA

**FIGURE 9:**
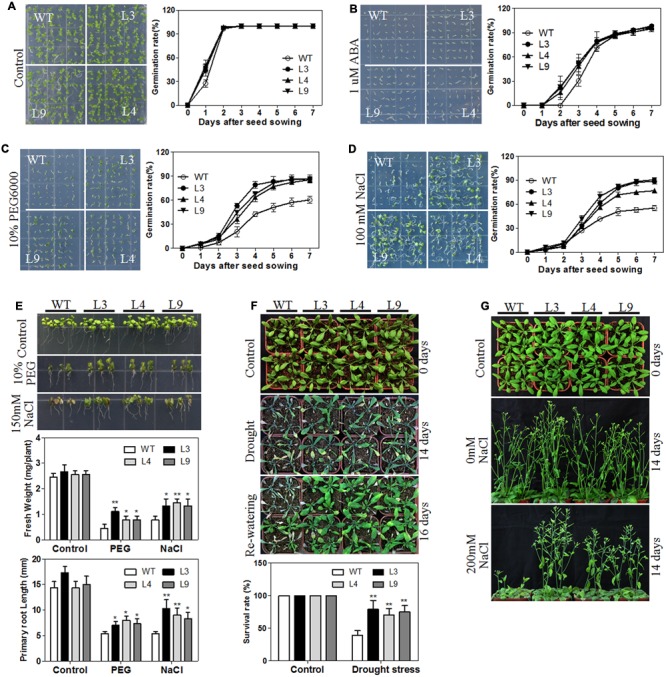
**Overexpression of *TaARGOS-D* confers drought and salt tolerance to transgenic *Arabidopsis* plants. (A–D)** Seed germination in WT and *TaARGOS-D* overexpressing plants. The picture was taken 7 days after sowing. **(E)** Images of 7-day-old seedlings of WT and three transgenic lines treated with 10% PEG6000 or 150 mM NaCl for 5 days. The fresh weight and root lengths of the seedlings were measured. **(F)** Phenotypes of WT and three transgenic lines during drought stress. The 4-week-old seedlings (0 days) of WT and transgenic lines were subjected to drought stress treatment by withholding irrigation for 14 days, after which watering was resumed and plants grew for another 2 days. The percentage of survived plants in drought stress was measured. **(G)** Images of 4-week-old, soil-grown plants (WT and three transgenic lines; 0 days) that were irrigated with water containing 0 or 200 mM NaCl every 3 days for 14 days. The results are means of three replicates ± S.D. Statistical significance was determined by a Student’s *t*-test; significant differences (*P* ≤ 0.05) are indicated by asterisk.

The plant hormone ABA mediates plant seed germination (Jones, 2016). We showed that the abundance of several ABA-related genes was altered in our RNA-seq data (Supplementary Table S3) and that the expression of the *TaARGOSs* was significantly induced by ABA (**Figure 4D**). To investigate whether the *TaARGOSs* are involved in the ABA-mediated inhibition of seed germination, we treated the homozygous transgenic lines and WT plants with exogenous ABA. As shown in **Figure 9A**, the germination rates of the transgenic lines and WT plants were similar on ABA-free medium after 2 days, but those of the transgenic lines were less affected on ABA-containing medium (**Figure 9B**). For example, after 3 days of germination, ∼50% of the transgenic seeds germinated, while only ∼30% of the WT seeds germinated. These results demonstrated that the transgenic lines were less sensitive to ABA compared with WT plants during seed germination and that *TaARGOS-D* negatively regulates the ABA-induced inhibition of seed germination.

### *TaARGOS-D* Transgenic Plants Exhibited Improved Tolerance to Salt and Drought Stresses

ABA has been demonstrated to be required for abiotic stress tolerance during seed germination and seedling growth (Zhu, 2002; Huang Y. et al., 2016). We next tested the germination rates of transgenic and WT plants under PEG or salt stress conditions. As shown in **Figures 9C,D**, the germination rates of the transgenic seeds were significantly higher than those of the WT seeds under PEG or NaCl treatment. Similarly sized WT and transgenic seedlings were then selected and planted vertically in either 0 or 10% PEG6000 or 150 mM NaCl MS medium for 5 days. As expected, no significant differences in fresh weight and root length were observed between the transgenic and WT plants under normal conditions. However, when grown in 10% PEG6000- or 150 mM NaCl-containing MS medium, the transgenic seedlings exhibited a significantly greater fresh weight and longer roots than the WT plants, and the transgenic seedlings were less prone to chlorosis (**Figure 9E**).

To assess the performance of the *TaARGOS-D* transgenic plants under drought or salt stress in soil, the growth of the transgenic and WT plants was tested under normal or stress conditions. As expected, the transgenic plants exhibited a WT phenotype under normal conditions. However, after 2 weeks of withholding water, all of the WT plants exhibited severe wilting, and some had died, while only some of the *TaARGOS-D* transgenic plants showed signs of severe dehydration, and the rosette leaves of some of the transgenic plants were still green and fully expanded (**Figure 9F**). After 2 days of re-watering, only 40% of the WT plants survived, whereas 80, 70, and 75% of the transgenic plants survived in lines L3, L4, and L9, respectively (**Figure 9F**). In addition, when treated with 200 mM NaCl for 14 days, the transgenic plants were able to maintain their growth, while the WT plants exhibited significantly inhibited growth or even began to die (**Figure 9G**). Therefore, we can conclude that the transgenic plants are more tolerant to drought and salt stresses compared with WT plants.

## Discussion

In this study, we isolated and characterized the *TaARGOSs*, which are the homologs of reported *ARGOS* genes in *Arabidopsis*, rice and maize and encode OSR family proteins. Multiple sequence alignments showed that the TaARGOS proteins contain a conserved OSR domain with an identical LPPLPPPP motif and two putative transmembrane helices (**Figure 2A**). It has been reported that the OSR domain is sufficient to promote organ growth (Feng et al., 2011), thereby implying that the *TaARGOSs* and other plant ARGOSs share similar functions. In addition, the TaARGOSs and their orthologous in diploid, tetraploid, and hexaploid wheat are highly conserved (**Figure 3**). This high degree of conservation implies that the *TaARGOSs* might play essential roles in growth and development and that any sequence variation has adverse effects on plant growth.

Promoter-GUS activity assays revealed that the *GUS* gene was mainly expressed in vascular tissue (**Figure 5B**). The vascular tissue runs through the stem and branches off to different parts of the panicle. The *TaARGOSs* may be involved in the transport of specific molecules to the panicle through the vascular conductive system. This hypothesis is consistent with the finding that the expression of the *TaARGOSs* was relatively high in the stem and developing spikes (**Figure 4A**). In addition, most hexaploid wheat genes are present as triplicate homeologs, and a large proportion of wheat homeologs exhibit expression partitioning (Liu et al., 2015). The triplicate homeologs have three possible evolutionary fates: retention of the original function, gene silencing, and functional diversification (Shitsukawa et al., 2007). For example, Shitsukawa et al. (2007) showed that the three homoeologs genes of *wheat LEAFY HULL STERILE1* (*WLHS1*) present genetic and epigenetic differences, and analyses of expression patterns and protein functions showed that only *WLHS1-D* is functional in hexaploid wheat. In the present study, different expression patterns were observed for the *TaARGOSs*; *TaARGOS-D* showed the highest expression level in all of the tested tissues, while *TaARGOS-B* was only highly expressed in the wheat stems and YS (**Figure 4A**). Generally, tissue-specific gene expression is associated with specific physiological and developmental functions (Zhang et al., 2012). Based on this, we speculated that *TaARGOS-B* may play a role in the development of stems and YS. In terms of *TaARGOS-A*, it might have similar functions with *TaARGOS-D* since they share similar tissue specific expression patterns and responses to hormones and stress treatments. These results indicated that *TaARGOSs* might have undergone functional diversification, the roles of *TaARGOS* homoeologous genes in wheat need to be further elucidated.

Similar to other identified OSR members, TaARGOS-D was localized to the ER (**Figure 6**). Triterpene genes thalianol synthase (THAS), marneral synthase (MRN1), and marneral oxidase (MRO) were previously shown to be ER-localized (Field et al., 2011). Interestingly, the expression levels of these genes were enhanced in the *TaARGOS-D* transgenic plants as revealed by RNA-seq analysis. Previous studies have shown that triterpenes protect plants against diseases and environmental stresses and are essential precursors for cell membranes and steroid hormones during plant growth and development (Field et al., 2011). Therefore, *TaARGOS-D* might function together with these molecules to affect the transgenic plants growth and development. In addition, the RNA-seq data also showed that in transgenic plants, four bHLH transcription factor genes (*bHLH38, bHLH39, bHLH100*, and *bHLH101*) and many seed-specific genes, including *At2S3, CRC, AtEm1*, and *TIP3-1*, were transcriptionally inhibited. Previous studies showed that the transcripts of the four *bHLH* genes were strongly up-regulated during early leaf development and were significantly decreased afterward (Wang et al., 2007; Andriankaja et al., 2012, 2014). The expression of seed-specific genes is gradually decreased during seedling growth (Herman and Larkins, 1999). Therefore, the decreased expression of bHLH and seed-specific genes in *TaARGOS-D* transgenic plants compared with that in WT indicated that the WT might be at an earlier development stage than the transgenic plants. In other words, the transgenic seedlings grew faster than the WT.

**FIGURE 10:**
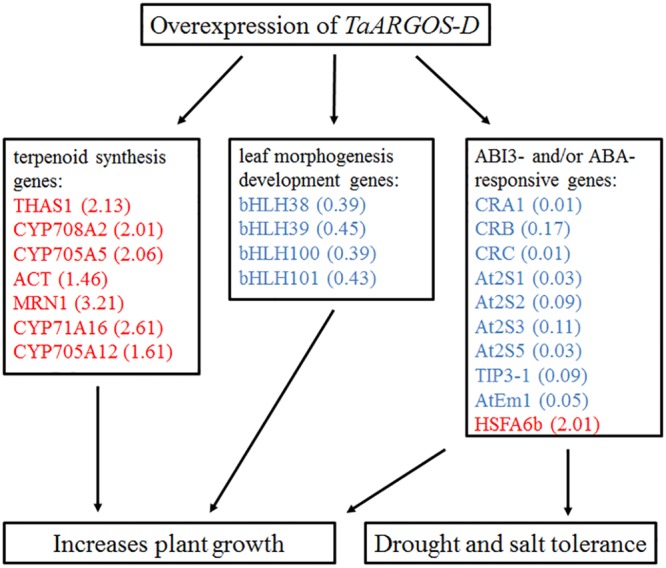
**Proposed model linking overexpression of *TaARGOS-D* and plant growth and stress tolerance.** Red color represents up-regulated genes and blue color represents down-regulated genes. The numbers in brackets indicate the fold change of transcripts.

The phytohormone ABA plays an important role in plant developmental processes such as seed germination, seedling growth, and stress tolerance (Leung and Giraudat, 1998; Rock, 2000; Verma et al., 2016). ABI3, which is a central regulator in ABA signaling, represses germination and seedling development (Parcy et al., 1994). It was revealed that *abi3* mutant markedly impaired the accumulation of genes *At2S3, CRC, AtEm1*, and *TIP3-1*, and the germinating seeds exhibited reduced sensitivity to exogenous ABA (Ooms et al., 1993; Parcy et al., 1994; Nakashima et al., 2006). In this study, the expression levels of the *TaARGOSs* were found to be increased in response to ABA treatment (**Figure 4D**) and overexpression of the *TaARGOS-D* genes decreased ABA sensitivity in seed germination (**Figure 9B**). Interestingly, *At2S3, CRC, AtEm1*, and *TIP3-1*, which were identified as the target genes of ABI3 (Parcy et al., 1994; Nakashima et al., 2006; Mao and Sun, 2015), were shown to be inhibited in *TaARGOS-D* transgenic seedlings. Taken together, we speculate that the phenotypes conferred by *TaARGOS-D* might be due to *TaARGOS-D*-mediated decreases in the expression of *ABI3* at the germination stage. In order to verify this hypothesis, we examined the expression of *ABI3* and its directly regulated genes (*At2S3, CRC*, and *TIP3-1*) at 2 days after the imbibition in overexpression lines and WT, and marked decrease of these genes were observed in overexpression lines (Supplementary Figure S4). A recent study demonstrated that *HSFA6b* played a pivotal role in ABA-mediated salt and drought resistance and that overexpression of *HSFA6b* enhanced drought, salt, and heat stress tolerance in transgenic *Arabidopsis* compared with control plants (Huang Y.C. et al., 2016). Intriguingly, the RNA-seq data also revealed that *HSFA6b* gene expression was up-regulated in *TaARGOS-D* transgenic plants. Taken together, our results suggest that overexpression of *TaARGOS-D* altered the expression levels of genes involved in the terpenoid synthesis and leaf morphogenesis development, and ABI3- and/or ABA-responsive genes, thereby enhancing plant growth and conferring salt and drought tolerance in *Arabidopsis* (**Figure 10**). Future genetic and molecular biological evidence is required.

## Author Contributions

YZ and XT contributed equally to this work. They carried out the experiments, analyzed the results, and wrote the manuscript. MX and HP designed the experiments. QS, ZN, YY, and ZH provided the idea, instructed the research work and revised the manuscript. YL, LZ, PG, XK, and XW provided assistance to perform experiments and collect data. All authors have read and approved the final manuscript.

## Conflict of Interest Statement

The authors declare that the research was conducted in the absence of any commercial or financial relationships that could be construed as a potential conflict of interest.

## Footnotes

1 http://www.wheatgenome.org/

2 http://www.uniprot.org/

3 http://www.bio-rad.com/

4 http://bioinfo.cau.edu.cn/agriGO/index.php

5 http://bioinformatics.psb.ugent.be/webtools/plantcare/html/
