# Supplementary material for: Molecular and Functional Characterization of Wheat ARGOS Genes Influencing Plant Growth and Stress Tolerance
Source: Front Plant Sci. 2017 Feb 8;8:170. doi: 10.3389/fpls.2017.00170 (PMC5296299; doi:10.3389/fpls.2017.00170)
Supplement: Supplementary file 1 [file Data_Sheet_1.DOC]

Supplementary Material

# Molecular and functional characterization of wheat *ARGOS* genes influencing plant growth and stress tolerance

Yue Zhao†, Xuejun Tian†, Yuanyuan Li, Liyuan Zhang, Panfeng Guan, Xiaoxia Kou, Xiaobo Wang, Mingming Xin, Zhaorong Hu, Yingyin Yao, Zhongfu Ni, Qixin Sun, Huiru Peng*

*Correspondence: Huiru Peng: [penghuiru@cau.edu.cn](mailto:penghuiru@cau.edu.cn)

# Supplementary Figures and Tables

**
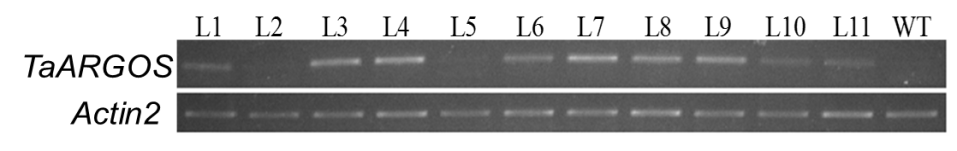
**

**Figure S1** RT-PCR analysis of 11 independent overexpression lines of *TaARGOS-D*; upper panel shows *TaARGOS-D* expression (34 cycles) in transgenic lines and WT; lower panel shows *Actin2* expression (25 cycles) as a control. L3, L4, and L9 lines were selected for further analysis.

**Figure S2** Overexpression lines of *TaARGOS-D* showed faster germination rates. The average germination percentages ±SD of triplicates were calculated.

**
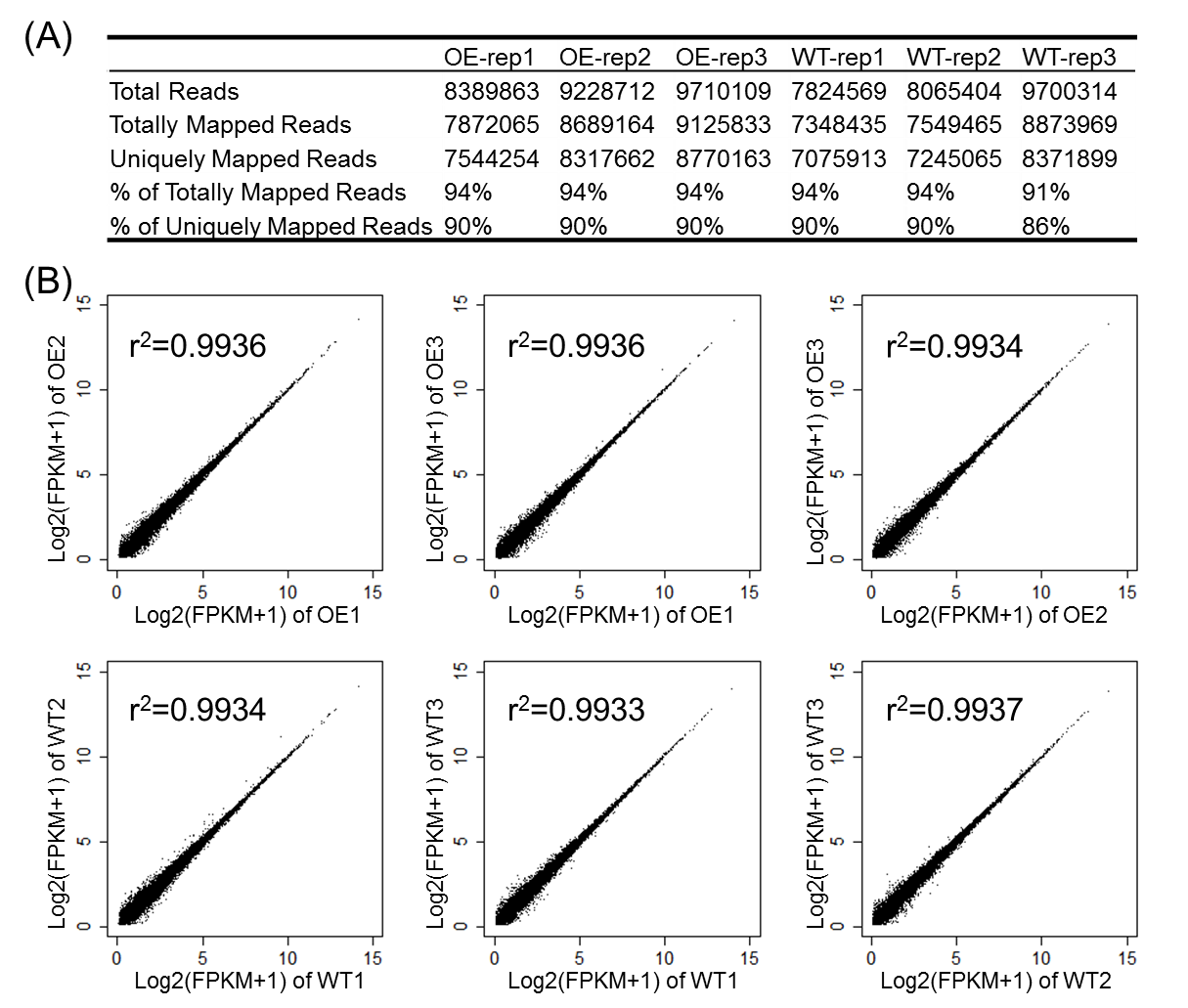
**

**Figure S3** RNA-seq data analysis. (A) A summary of read counts. (B) Correlation assessment of a pair of biological replicates in WT and *TaARGOS-D* transgenic plants, respectively. Each dot in the plot represents a gene, denoting the log_2_ of the gene expression (FPKM) in the two replicate samples (x-axis and y-axis).

**Figure S4** *Arabidopsis* *ABI3* and its directly regulate gene (*At2S3*, *CRC*, and *TIP3-1*) expression in *TaARGOS-D* overexpression lines and WT. Total RNA was isolated from seeds at 2 days after the imbibition. The *Arabidopsis Actin2* gene was used as an internal reference. Mean and standard deviation were calculated from three independent biological replicates.

**Table S1.** Plant materials of the *Triticum* and *Aegilops* accessions used in this study.

| No. | Materials accession no. | Species | Genome | Origin |
| --- | --- | --- | --- | --- |
| 1 | KU-199-1 | *T. urartu* | A | USSR |
| 2 | KU-199-4 | *T. urartu* | A | USSR |
| 3 | KU-199-6 | *T. urartu* | A | Lebanon |
| 4 | PI 542238 | *Ae. speltoides* | S | Turkey |
| 5 | PI 554304 | *Ae. speltoides* | S | Turkey |
| 6 | PI 542251 | *Ae. speltoides* | S | Turkey |
| 7 | AS84 | *Ae. tauschii* | D | Iran |
| 8 | AS90 | *Ae. tauschii* | D | Iran |
| 9 | AS92 | *Ae. tauschii* | D | Iran |
| 10 | AS641009 | *T. dicoccum* | AB | Georgia |
| 11 | AS641013 | *T. dicoccum* | AB | Serbia |
| 12 | AS641017 | *T. dicoccum* | AB | United States |
| 13 | Chinese Spring | *T. aestivum* | ABD | China |
| 14 | Jingdong6 | *T. aestivum* | ABD | China |
| 15 | Nongda3338 | *T. aestivum* | ABD | China |
| 16 | Zang1817 | *T. aestivum* | ABD | China |
| 17 | Kenong199 | *T. aestivum* | ABD | China |
| 18 | Ji5265 | *T. aestivum* | ABD | China |

KU, Plant Germ-Plasm Institute, Faculty of Agriculture, Kyoto University, Japan.

PI, National Small Grains Research Facility, USDA-ARS, USA.

AS, Triticeae Research Institute of Sichuan Agriculture University

**Table S2.** Primer sequences used in this study.

| Reactions | Primer name | Sequence information | Enzyme  site | |
| --- | --- | --- | --- | --- |
| Genomic | TaARGOS-AF1 | CGCTAAGCTTTGCATTTCTGAC |  | |
| DNA | TaARGOS-AR1 | GCACTCATCTTATGCCAACAC |  | |
|  | TaARGOS-BF1  TaARGOS-BR1  TaARGOS-DF1  TaARGOS-DR1 | ATTTACACCACTAATTGGGTCG  GCACTCATCTTATGCCAACAC  TTACACCACTACTTGGGTCAGC  GCACTCATCTTATGCCAACAC |  | |
| cDNA | TaARGOS-AF2 | CCGTAAACTAATCCCGTTTGGT |  | |
|  | TaARGOS-AR2 | GCACTCATCTTATGCCAACAC |  | |
|  | TaARGOS-BF2  TaARGOS-BR2  TaARGOS-DF2  TaARGOS-DR2 | CCGTAAACTAATCCCATTTCGT  GCACTCATCTTATGCCAACAC  CGTAAACTAATCCCGTTTCGC  GCACTCATCTTATGCCAACAC |  | |
| qRT-PCR | TaARGOS-qAF | CTCGCCTGCCTTACCGTA |  | |
|  | TaARGOS-qAR | GCCATGCTCCGCATATCC |  | |
|  | TaARGOS-qBF | TGATTTGGAGGAGAGGGTGTT |  | |
|  | TaARGOS-qBR | GCCCTCTGCTGCTGCATA |  | |
|  | TaARGOS-qDF | GCTGATCTGCACTCACCAAAC |  | |
|  | TaARGOS-qDR | AACAGCAGCAGCGAAGGC |  | |
|  | β-action-F | GGAATCCATGAGACCACCTAC |  | |
|  | β-action-R | GACCCAGACAACTCGCAAC |  | |
| RT-PCR | TaARGOS-RTF | TCATTGCTCATACTGCCATTG |  | |
|  | TaARGOS-RTR | GCACTCATCTTATGCCAACAC |  | |
|  | Actin2-F | GCTCCTCTTAACCCAAAGGC |  | |
|  | Actin2-R | CACACCATCACCAGAATCCAGC |  | |
| Subcellular localization | TaARGOS-SF | AGCCCAAGCTTATGGATAGCCAGTTCGGT | Hind III | |
|  | TaARGOS-SR | GCTCTAGACAAGTAAGAGGTGGCCAT | Xba I |  |
| GUS expression | TaARGOS-GDF | TCCCCCGGGTTACACCACTACTTGGGTC | Sma I | |
|  | TaARGOS-GDR | TAACTGCAGCTATTGTTTGGTGAGTGCAG | Pst I |  |
| Transgenic | TaARGOS-TDF | GGGGACAAGTTTGTACAAAAAAGCAGGCTTCATGGATAGCCAGTTCGGT | attB site | |
| *Arabidopsis* | TaARGOS-TDR | GGGGACCACTTTGTACAAGAAAGCTGGGTCTTACAAGTAAGAGGTGG | attB site | |
| RNA-seq | TIE1-F | TACCTACCCTTCTTATCATCCCT |  | |
| analysis | TIE1-R | GAGGCTGAGAATCTCCGTATCTT |  | |
|  | MRN1-F | GTTAGACAAAGAGCACTACGAACC |  | |
|  | MRN1-R | TACCTCAAGGTACTCTTGCTCC |  | |
|  | WRKY62-F | GCACATATGCCAGCTTCATC |  | |
|  | WRKY62-R | TGGGATGGAGACCAATCTTC |  | |
|  | TIP3-F | CCCACCGAACCACCTACC |  | |
|  | TIP3-R | GAACAACGAACAAAAGCA |  | |
|  | At2S3-F | AGCAAAACATGGCTAACAAGCTCT |  | |
|  | At2S3-R | CTGGCATCTCTGTCTTGGACCT |  | |
|  | CRC-F | CAGCCACTTGTCATCATCG |  | |
|  | CRC-R | TAGGAAGCACCATCGCATTA |  | |
|  | CRA1-F | CACGGTAATGGCTTAGAGGA |  | |
|  | CRA1-R | ACTGGAATCGGTTGCTTGTT |  | |
|  | CRB-F | GCCATCACTCGGATACATTA |  | |
|  | CRB-R | GGTCTCAATCGTGCTAAACT |  | |
|  | ABI3-F | TCCATTAGACAGCAGTCAAGGTTT |  | |
|  | ABI3-R | GGTGTCAAAGAACTCGTTGCTATC |  | |

**Table S3. List of assembled transcripts that were up-regulated or down-regulated (fold change (FC) > 2 or < 0.5, FDR < 0.05) in *TaARGOS-D* transgenic *Arabidopsis* plants relative to WT plants.**

| Gene_ID | WT | OE | FC | P-value | FDR | Profile | Gene_Name |
| --- | --- | --- | --- | --- | --- | --- | --- |
| AT4G28520.1 | 15.22±2.05 | 0 | 0.004 | 3.9E-150 | 4.4E-146 | down | CRC |
| AT5G44120.3 | 21.86±2.68 | 0.21±0.08 | 0.013 | 6.5E-186 | 1.5E-181 | down | CRA1 |
| AT2G27380.1 | 1.78±0.51 | 0 | 0.029 | 9.73E-26 | 6.64E-23 | down | EPR1 |
| AT5G54740.1 | 5.81±0.85 | 0.05±0.08 | 0.032 | 1.23E-35 | 1.54E-32 | down | SESA5 |
| AT4G27140.1 | 5.13±1.03 | 0 | 0.033 | 5.71E-26 | 4.15E-23 | down | AT2S1 |
| AT1G68250.1 | 3.22±0.03 | 0 | 0.044 | 1.09E-19 | 4.72E-17 | down |  |
| AT3G51810.1 | 2.55±0.49 | 0 | 0.050 | 2.79E-17 | 9.52E-15 | down | EM1 |
| AT2G28490.1 | 2.03±0.69 | 0.04±0.04 | 0.053 | 7.79E-21 | 3.58E-18 | down |  |
| AT5G40420.1 | 3.9±0.91 | 0.09±0.08 | 0.057 | 1.58E-22 | 8.45E-20 | down | OLEO2 |
| AT3G54940.2 | 1.25±0.26 | 0 | 0.068 | 9.12E-13 | 1.96E-10 | down |  |
| AT3G17520.1 | 2.29±0.47 | 0.07±0.06 | 0.075 | 5.4E-17 | 1.82E-14 | down |  |
| AT4G25140.1 | 3.98±0.84 | 0.18±0.15 | 0.075 | 7.12E-22 | 3.73E-19 | down | OLEO1 |
| AT3G22640.1 | 1.52±0.56 | 0.05±0.04 | 0.077 | 8.17E-14 | 2.02E-11 | down | PAP85 |
| AT1G73190.1 | 2.17±0.31 | 0.1±0.01 | 0.088 | 2.27E-16 | 7.2E-14 | down | TIP3-1 |
| AT4G27150.1 | 3.34±0.77 | 0.15±0.15 | 0.090 | 1.26E-15 | 3.72E-13 | down | AT2S2 |
| AT4G21020.1 | 1.12±0.3 | 0 | 0.093 | 3.73E-09 | 4.59E-07 | down |  |
| AT1G48130.1 | 3.83±0.82 | 0.27±0.05 | 0.100 | 5.59E-20 | 2.52E-17 | down | PER1 |
| AT4G27160.1 | 1.34±0.13 | 0 | 0.106 | 5.97E-08 | 5.72E-06 | down | AT2S3 |
| AT1G04560.1 | 1.19±0.6 | 0 | 0.107 | 1.69E-07 | 1.44E-05 | down |  |
| AT4G09600.1 | 3.2±0.83 | 0.19±0.33 | 0.116 | 7.93E-12 | 1.48E-09 | down | GASA3 |
| AT3G15670.1 | 2.34±0.36 | 0.19±0.12 | 0.124 | 2.6E-13 | 6.05E-11 | down |  |
| AT1G54870.1 | 0.9±0.29 | 0.03±0.05 | 0.127 | 2.24E-07 | 1.86E-05 | down |  |
| AT2G21490.1 | 0.99±0.4 | 0 | 0.129 | 2.22E-06 | 0.000136 | down | LEA |
| AT2G35300.1 | 6.46±2.17 | 0.63±0.36 | 0.131 | 1.79E-13 | 4.24E-11 | down | LEA18 |
| AT3G56350.1 | 0.74±0.36 | 0 | 0.150 | 1.87E-05 | 0.000853 | down | MSD2 |
| AT5G44310.2 | 0.71±0.25 | 0.03±0.05 | 0.152 | 3.19E-06 | 0.00019 | down |  |
| AT3G21380.1 | 0.85±0.08 | 0.07±0.07 | 0.157 | 1.23E-07 | 1.1E-05 | down | JAL36 |
| AT3G21370.1 | 1.21±0.44 | 0.14±0.09 | 0.161 | 8.29E-10 | 1.13E-07 | down | BGLU19 |
| AT2G41260.2 | 2.14±0.09 | 0.25±0.08 | 0.161 | 2.98E-11 | 4.91E-09 | down | M17 |
| AT2G42560.1 | 0.37±0.05 | 0.02±0.03 | 0.170 | 2.1E-05 | 0.00094 | down |  |
| AT1G03880.1 | 0.36±0.12 | 0 | 0.171 | 0.000122 | 0.003838 | down | CRB |
| AT5G22470.1 | 0.2±0.05 | 0 | 0.181 | 0.000244 | 0.006614 | down | PARP3 |
| AT5G55410.2 | 0.94±0.61 | 0.06±0.1 | 0.206 | 0.000429 | 0.01026 | down |  |
| AT1G75830.1 | 1.5±0.59 | 0.09±0.15 | 0.208 | 0.000281 | 0.00739 | down | PDF1.1 |
| AT3G53040.1 | 0.75±0.35 | 0.1±0.12 | 0.210 | 1.98E-05 | 0.000895 | down |  |
| AT1G72100.1 | 0.39±0.28 | 0.03±0.04 | 0.214 | 0.000518 | 0.012008 | down |  |
| AT4G36700.1 | 3.36±0.25 | 0.67±0.25 | 0.215 | 4.33E-21 | 2.17E-18 | down |  |
| AT3G50980.1 | 0.67±0.34 | 0 | 0.220 | 0.001995 | 0.034498 | down | XERO1 |
| AT3G02480.1 | 23.12±2.24 | 6.2±1.96 | 0.277 | 4.46E-25 | 2.87E-22 | down |  |
| AT4G33720.1 | 1.1±0.44 | 0.2±0.18 | 0.288 | 0.000989 | 0.02002 | down |  |
| AT1G05510.1 | 0.69±0.13 | 0.12±0.11 | 0.295 | 0.002577 | 0.041431 | down |  |
| AT1G52690.1 | 1.69±0.4 | 0.4±0.15 | 0.297 | 4.2E-05 | 0.00166 | down |  |
| AT1G52120.1 | 1.17±0.09 | 0.3±0.15 | 0.299 | 5.39E-06 | 0.000298 | down | JAL12 |
| AT4G04223.1 | 8.51±0.89 | 2.44±0.13 | 0.305 | 5.47E-12 | 1.05E-09 | down |  |
| AT5G52300.1 | 0.69±0.23 | 0.18±0.02 | 0.318 | 7.29E-05 | 0.002547 | down | LTI65 |
| AT4G20160.1 | 0.24±0.06 | 0.05±0.02 | 0.318 | 0.000911 | 0.018712 | down |  |
| AT2G47050.1 | 1.46±0.52 | 0.4±0.39 | 0.330 | 0.000377 | 0.009268 | down |  |
| AT4G27570.1 | 1.17±0.28 | 0.34±0.14 | 0.333 | 1.92E-05 | 0.00087 | down | UGT79B3 |
| AT5G40590.1 | 3.45±0.48 | 1.12±0.6 | 0.347 | 8.52E-08 | 7.9E-06 | down |  |
| AT5G07700.1 | 1.01±0.15 | 0.3±0.11 | 0.370 | 0.002103 | 0.035666 | down | MYB76 |
| AT2G14247.1 | 61.81±3.44 | 23.27±3.18 | 0.379 | 5.11E-45 | 9.6E-42 | down |  |
| AT5G35935.1 | 0.28±0.1 | 0.1±0.02 | 0.391 | 0.000404 | 0.009755 | down |  |
| AT2G41240.1 | 61.88±2.68 | 24.13±3.65 | 0.392 | 3.35E-54 | 1.08E-50 | down | BHLH100 |
| AT3G06710.1 | 0.77±0.12 | 0.25±0.05 | 0.395 | 0.002564 | 0.04131 | down |  |
| AT3G56970.1 | 33.23±1.7 | 13.05±0.7 | 0.395 | 2.58E-46 | 7.26E-43 | down | BHLH38 |
| AT1G10585.1 | 2.48±0.4 | 0.91±0.35 | 0.407 | 0.000337 | 0.008479 | down |  |
| ATMG01170.1 | 1.86±0.24 | 0.72±0.07 | 0.414 | 3.49E-05 | 0.001431 | down | ATP6-2 |
| AT5G19890.1 | 3.48±0.23 | 1.39±0.49 | 0.416 | 1.05E-07 | 9.59E-06 | down | PER59 |
| AT5G04150.1 | 13.69±1.37 | 5.87±0.74 | 0.436 | 5.09E-14 | 1.29E-11 | down | BHLH101 |
| AT3G01570.1 | 2.54±0.89 | 1.04±0.05 | 0.440 | 0.000481 | 0.011261 | down |  |
| AT3G56980.1 | 38.98±1.06 | 17.28±0.26 | 0.445 | 1.04E-40 | 1.67E-37 | down | BHLH39 |
| AT3G21720.1 | 19.59±0.22 | 8.83±1.14 | 0.452 | 4.99E-45 | 9.6E-42 | down | ICL |
| AT2G37530.1 | 2.7±0.87 | 1.19±0.72 | 0.474 | 0.003071 | 0.046863 | down |  |
| AT1G13609.1 | 57.29±5.15 | 27.03±3.46 | 0.475 | 5.06E-21 | 2.48E-18 | down |  |
| AT1G47395.1 | 132.37±7.17 | 63.29±2.59 | 0.479 | 7.17E-45 | 1.24E-41 | down |  |
| AT4G31940.1 | 17.76±1.41 | 8.64±0.44 | 0.489 | 3.13E-29 | 2.94E-26 | down | CYP82C4 |
| AT5G37400.1 | 0.95±0.17 | 0.44±0.22 | 0.492 | 0.002188 | 0.036748 | down |  |
| AT4G15690.1 | 3.61±1.2 | 1.68±0.83 | 0.493 | 0.003288 | 0.049369 | down | GRXS5 |
| AT1G47400.1 | 42.05±2.77 | 20.72±2.86 | 0.496 | 2.85E-19 | 1.19E-16 | down |  |
| AT2G01505.1 | 4.93±1.23 | 2.34±0.2 | 0.499 | 0.00117 | 0.022992 | down | CLE16 |
| AT3G22830.1 | 1.04±0.33 | 2.16±0.71 | 2.006 | 0.000973 | 0.019786 | up | HSFA6B |
| AT5G48000.1 | 11.21±0.2 | 22.59±1.1 | 2.010 | 1.99E-37 | 2.8E-34 | up | CYP708A2 |
| AT2G04460.1 | 2.42±0.65 | 4.96±0.61 | 2.016 | 9.38E-08 | 8.56E-06 | up |  |
| AT5G47990.1 | 13.76±0.86 | 28.44±0.96 | 2.061 | 1.51E-45 | 3.4E-42 | up | CYP705A5 |
| AT4G37070.2 | 5.53±0.39 | 11.53±1.2 | 2.071 | 8.37E-17 | 2.73E-14 | up | PLP1 |
| AT1G52030.2 | 0.52±0.07 | 1.15±0.21 | 2.100 | 0.000214 | 0.005997 | up | F-ATMBP |
| AT2G14560.1 | 7.1±0.95 | 15.07±0.95 | 2.109 | 1.3E-23 | 7.7E-21 | up | LURP1 |
| AT5G48010.2 | 11.36±0.68 | 24.25±0.64 | 2.130 | 2.23E-56 | 8.38E-53 | up | THAS1 |
| AT3G62740.1 | 0.92±0.33 | 2.09±0.45 | 2.165 | 0.000106 | 0.003453 | up | BGLU7 |
| AT1G77520.1 | 1.16±0.17 | 2.69±0.44 | 2.234 | 4.17E-06 | 0.000241 | up |  |
| AT3G44326.1 | 1.04±0.33 | 2.47±0.28 | 2.249 | 2.77E-05 | 0.001186 | up |  |
| AT2G47530.1 | 1.39±0.17 | 3.38±1.76 | 2.267 | 0.002091 | 0.035514 | up |  |
| AT4G28840.1 | 1.5±0.47 | 3.62±1.79 | 2.282 | 0.00143 | 0.026767 | up | TIE1 |
| AT1G13520.1 | 0.64±0.1 | 1.6±0.16 | 2.300 | 0.000536 | 0.012355 | up |  |
| AT1G66100.1 | 9.02±0.52 | 21.22±0.35 | 2.327 | 9.95E-20 | 4.4E-17 | up |  |
| AT4G14060.1 | 2.65±0.58 | 6.46±0.87 | 2.348 | 3.74E-07 | 2.9E-05 | up |  |
| AT5G66640.2 | 0.44±0.25 | 1.15±0.29 | 2.367 | 0.000617 | 0.013762 | up | DAR3 |
| AT5G23980.1 | 0.25±0.05 | 0.66±0.18 | 2.388 | 0.00177 | 0.031541 | up | FRO4 |
| AT3G21340.1 | 0.24±0.13 | 0.67±0.04 | 2.473 | 0.000337 | 0.008479 | up |  |
| AT3G44716.1 | 0.86±0.15 | 2.46±0.9 | 2.489 | 0.001661 | 0.029987 | up |  |
| AT4G31970.1 | 0.68±0.14 | 1.83±0.06 | 2.508 | 1.01E-05 | 0.000518 | up | CYP82C2 |
| AT1G77530.1 | 0.61±0.15 | 1.68±0.58 | 2.523 | 0.000288 | 0.007525 | up |  |
| AT4G35650.1 | 0.31±0.08 | 0.94±0.04 | 2.577 | 0.001915 | 0.033524 | up | IDH3 |
| AT3G48080.1 | 0.35±0.14 | 1±0.22 | 2.607 | 0.000112 | 0.003621 | up | EDS1B |
| AT5G42590.1 | 1.15±0.18 | 3.11±0.18 | 2.611 | 1.18E-09 | 1.51E-07 | up | CYP71A16 |
| AT3G20460.1 | 0.56±0.46 | 1.61±0.22 | 2.626 | 6.01E-05 | 0.002215 | up |  |
| AT2G14610.1 | 1.41±0.6 | 4.05±0.5 | 2.683 | 1.45E-06 | 9.48E-05 | up | PR1 |
| AT3G23120.1 | 0.18±0.1 | 0.57±0.19 | 2.688 | 0.001479 | 0.027428 | up | AtRLP38 |
| AT4G22610.1 | 0.94±0.6 | 2.93±0.33 | 2.695 | 0.00041 | 0.00988 | up |  |
| AT5G42600.1 | 0.15±0.06 | 0.59±0.23 | 3.214 | 0.000115 | 0.003673 | up | MRN1 |
| AT1G61080.1 | 0.08±0.07 | 0.36±0.08 | 3.388 | 0.000325 | 0.008259 | up |  |
| AT4G13280.1 | 0.21±0.06 | 0.95±0.27 | 3.561 | 2.49E-05 | 0.001089 | up | ATTPS12 |
| AT1G14880.1 | 0.55±0.19 | 2.4±0.95 | 3.591 | 1.22E-05 | 0.000599 | up | PCR1 |
| AT5G01900.1 | 0.05±0.08 | 0.55±0.22 | 3.930 | 0.001847 | 0.032591 | up | WRKY62 |
| AT3G51360.1 | 0.03±0.05 | 0.39±0.3 | 4.308 | 0.001609 | 0.029197 | up |  |
